# Supplementary material for: Drug–food and drug–alcohol interactions: Pharmacists’ knowledge gaps and patient safety concerns in the United Arab Emirates
Source: PLoS One. 2026 Mar 27;21(3):e0346144. doi: 10.1371/journal.pone.0346144 (PMC13028502; doi:10.1371/journal.pone.0346144)
Supplement: S1 Questionnaire — (PDF) [file pone.0346144.s001.pdf]

Food-drug interactions: Knowledge among community pharmacists in United Arab Emirates (UAE)

(Questionnaire)

Do you agree to participate in this study and to use the information for scientific research purposes?

□Yes

□No

Part (1). Demographics and general questions:

- Age group:

20-29 years

30-39 years

40-49 years

50-59 years

>60 years

Emirates:

Abu Dhabi

Ajman

Dubai

Ras Al Khaimah

Sharjah

Umm Al-Quwaim

Nationality

………………..

- Gender:

□ Male □ Female

- Education level:

 Bachelor of pharmacy Doctor of Pharmacy (Pharm D) Postgraduate studies

Where do you practice?

Chain Pharmacy

Individual Pharmacy

How many years have you been practicing?
≥ 2 years

3-5 years

6-10 years

11-15 years

>15 years

Does your pharmacy offer teleservices

No

Yes

No. of licensed staff per shift in the pharmacy

 3 or less

More than 3

Do you think you have enough information about food-drug interactions?

No

Yes

In your workplace, is it mandatory to attend conferences, workshops, seminars or course related to drug-food interaction?

No

Yes

During your career years, number of workshops, seminars, conferences or courses you attend related to drug food interaction.

……………………………..

- Which of the following age groups is most susceptible to food-drug interactions?

☐ Children ☐ Adults ☐ Elderly

- What is the main source for your knowledge of food-drug interactions?

☐ Publish Literature

☐ Conferences courses and workshop

☐ Consultation with peers and medical experts

☐ University Education

Part (2). Drugs-Food Interactions knowledge

1. Can amiodarone be taken with grapefruit?

   ☐ Yes ☐ No

2. Can atorvastatin be taken with grapefruit?

   ☐ Yes ☐ No

3. Does cauliflower consumption affect the efficacy of levothyroxine?

   ☐ Yes ☐ No

4. Does caffeine consumption affect the efficacy of diazepam?

   ☐ Yes ☐ No

5. Patients can eat more leafy green vegetables with Coumadin (warfarin):

   ☐ Yes ☐ No

6. Patient taking theophylline should avoid excessive coffee and tea:

   ☐ Yes ☐ No

7. Does milk affect the efficacy of tetracycline?

   ☐ Yes ☐ No

8. Patients taking monoamine oxidase inhibitors (MAOIs) should avoid eating aged cheeses:

□ Yes □ No

9. Does wheat bran diet affect the efficacy of digoxin?

□ Yes □ No

10. Does protein-rich foods affect the efficacy of levodopa?

□ Yes □ No

11. Grapefruit juice can be safely consumed with all antibiotics:

□ Yes □ No

12. Patients should avoid taking spironolactone with food rich in potassium?

□ Yes □ No

Part (3). Knowledge about timing of drug intake with respect to food:

Please choose the best time to take each medication with respect to food

	Medication	Before meal with 1/2 hour	With meal	Two hours after meal	Can be taken without regard to food
13.	Carbamazepine				
14.	Methotrexate				
15.	Isotretinoin				
16.	Omeprazole				
17.	Glipizide				
18.	NSAIDs				
19.	Levothyroxine				

20.	Griseofulvin				
21.	Metformin				
22.	Calcium carbonate supplement				
23.	Erythromycin stearate				
24.	Propranolol				

Part (4). Knowledge about Drugs-alcohol interactions:

For each medication, please choose whether there is drug-alcohol interaction or not ("Yes" means **there is** drug-alcohol interaction, "No" means **there is no** drug-alcohol interaction.

	Medication	Yes	NO
25.	Antihistamine		
26.	Paracetamol		
27.	Metformin		
28.	Isoniazid		
29.	Warfarin		
30.	Methotrexate		
