# Supplementary material for: Drug–food and drug–alcohol interactions: Pharmacists’ knowledge gaps and patient safety concerns in the United Arab Emirates
Source: PLoS One. 2026 Mar 27;21(3):e0346144. doi: 10.1371/journal.pone.0346144 (PMC13028502; doi:10.1371/journal.pone.0346144)
Supplement: Attachment — Submitted filename: Response to Reviewers.docx [file pone.0346144.s005.docx]

Dear Academic Editor and Reviewers,

We would like to thank the Academic Editor and reviewers for their valuable comments and suggestions. We addressed all points and revised the script accordingly. All changes are highlighted in the tracked-changes version of the manuscript. Our point-by-point responses are provided below.

We hope that this revision is satisfactory. Thank you.

| **Academic Editor Comments**   1. Please clarify the age-related findings. In univariate analysis, age was not significant (p = 0.232), but in multiple regression age 20–29 years became significant (p = 0.041). That can happen after adjustment, but the manuscript does not explain it. Since this is a borderline result, it needs cautious interpretation. 2. Please strengthen the limitation on sampling bias. The manuscript acknowledges possible selection bias from online recruitment, but this is later minimized. Please keep the limitation wording balanced and transparent.   **Reviewer #2:**   1. Alcohol use prevalence is required to be added in the introduction. 2. English proofreading is required.   . | ***Authors’ response:***  **Academic Editor: Thank you very much for your comments.**   1. We have clarified this point in the Discussion section and added an explanation that the association became significant after adjustment for other variables in the multivariable regression model. We added new sentences to the discussion read as “Although age was not significantly associated with knowledge scores in the univariate analysis, it became significant in the multivariable regression model after adjustment for other demographic variables. This suggests that potential confounding factors may influence the relationship between age and knowledge scores. However, given the borderline statistical significance, this finding should be interpreted with caution.” 2. We have revised the limitations section to present the potential sampling bias more transparently. The sentence: “however, as pharmacists are increasingly using smartphones and the internet in their work, this is unlikely to have had an impact on our findings.” Deleted and replaced with: “Third, the study may have been more susceptible to selection and recall biases due to the use of an online survey, which may have influenced the representativeness of the sample.”   **Reviewer #2: Thank you very much for your comments.**  1-“Although alcohol consumption in the United Arab Emirates is regulated, evidence indicates that alcohol use remains present and represents a public health concern in the country [7], underscoring the importance of awareness of potential drug–alcohol interactions in clinical practice.” Added to the introduction to address the comment.  2- Proofreading is done. Examples are below:  **Introduction**   - **Current**   For example, Interactions between alcohol and antihistamines, as well as nonsteroidal anti-inflammatory medicines (NSAIDs), elevate the risk of internal bleeding, and respiratory difficulties. or cause life-threatening cardiovascular complications when taken with monoamine oxidase inhibitors (MAOIs).  **Replaced with:**  For example, interactions between alcohol and antihistamines or nonsteroidal anti-inflammatory drugs (NSAIDs) may increase the risk of internal bleeding and respiratory complications. Alcohol may also cause life-threatening cardiovascular complications when combined with monoamine oxidase inhibitors (MAOIs).   - **Current**   medication review [13]According to international guidelines  **Space added**  medication review [13]. According to international guidelines   - **Current**   While excessive intake of vitamin K–rich foods diminish warfarin's safety and effectiveness  Replaced with  Excessive intake of vitamin K–rich foods **diminishes** warfarin's safety and effectiveness  **Results**   - **Current**   "Does protein-rich foods affect the efficacy of Levodopa?"  Replaced with  "Do protein-rich foods affect the efficacy of levodopa?"  **Discussion**   - **Current**   The results of this study revealed that pharmacists in the UAE have overall moderate knowledge among UAE pharmacists.  Replaced with:  The results of this study indicate that community pharmacists in the UAE have an overall moderate level of knowledge.   - **Current**   This is consistent with previous research that **demonstrate**  Replaced with:  This is consistent with previous research that **demonstrates**   - **Current**   UAE regarding drug interactions  To read better:  UAE regarding **drug–food and drug–alcohol interactions**  **Limitations**   - **Current**   second, the reliance on self-reported responses  Replaced with:  Second, the reliance on self-reported responses  **Future research**   - **Current**   lastly, investigating patients' knowledge  Replaced with:  **Lastly**, investigating patients' knowledge |
| --- | --- |
